# Trends in epilepsy mortality of three incidence cohorts across 2006–2023 in Sweden: a matched register-based study

**DOI:** 10.1016/j.lanepe.2025.101388

**Published:** 2025-07-23

**Authors:** Johan Zelano, André Idegård, David Larsson

**Affiliations:** a Department of Clinical Neuroscience, Institute of Neuroscience and Physiology, The Sahlgrenska Academy at the University of Gothenburg, Gothenburg, Sweden; b Department of Neurology, Sahlgrenska University Hospital, Member of ERN Epicare, Gothenburg, Sweden; c Wallenberg Center of Molecular and Translational Medicine, Gothenburg University, Sweden

**Keywords:** Seizures, Death, Treatment

## Abstract

**Background:**

Idiopathic epilepsy alone accounts for a large health burden as shown in the Global Burden of Diseases Study. In countries with ageing populations, secondary epilepsies are now even more common. Altered clinical practice and reduced use of older enzyme-inducing drugs may be beneficial, but understanding of trends in the prognosis of all epilepsy is needed. Our objective was to determine and study trends in mortality in persons with epilepsy in Sweden through 2006–2023.

**Methods:**

We performed a matched cohort study by cross-referencing National Patient, Drug, and Cause of Death Registers. We included all persons with a diagnosis of epilepsy and antiseizure medication (ASM) after 2006 (n = 61,375) and three age-/sex-matched comparators/case. Mortality was assessed for three incidence cohorts; 2006–2010, 2011–2015, and 2016–2020, totally and in four subgroups: age >50, vascular disease, generalized epilepsy, and age <20. Risk of death was assessed by Cox regression.

**Findings:**

Carbamazepine and valproic acid were common first ASMs in 2006–2010, but replaced by levetiracetam by 2016–2020. Valproate became less common in generalized epilepsy. The adjusted hazard ratio [HR] for death was 1.99 (95% confidence interval [CI]:1.90–2.08) in 2006–2010 and 1.90 (95% CI: 1.82–1.99) in 2016–2020. The adjusted HR for death was 1.59 (95% CI: 1.50–1.68) for persons with cardiovascular disease versus comparators during 2016–2020. A sensitivity analysis showed that the excces risk of cardiovascular death had decreased between our cohorts. Young persons with epilepsy had a 30–50 fold increased HR of death. Dementia and vascular disease were important risk factors for death in persons with epilepsy.

**Interpretation:**

Mortality in epilepsy has remained largely unchanged relative to age- and sex matched comparators. The increased use of non-inducing ASMs may have reduced vascular risk slightly. Efforts should be targeted to specific patient groups, particularly regarding epilepsy management in the young and vascular and neurodegenerative comorbidities in older persons with epilepsy.

**Funding:**

10.13039/501100004359Swedish Research Council, Swedish State through the ALF agreement, Knut and Ragnvi Jacobsson foundation.

**Research in context**

**Evidence before this study**

The reference list of a 2023 systematic review based on a PubMed/EMBASE search covering 1980–2022 was considered before this study. The authors concluded that high-quality epilepsy care was associated with lower mortality, but that a considerable knowledge gap exists on the impact of co-morbidities. The Global Burden of Disease Study found reduced global mortality from idiopathic epilepsy, but with ageing populations the prevalence of non-idiopathic (secondary/structural) epilepsies increases. In another reference from Denmark, mortality for all epilepsy had remained unchanged between 2010 and 2015 and that life expectancy was reduced by 14 years in symptomatic epilepsy, exceeding the 8-10 years in idiopathic epilepsy. Since 2015, newer antiseizure medications (ASMs) with less negative effects on vascular health and fewer interaction with cardiovascular drugs have become more popular. We performed a detailed analysis of how epilepsy mortality has changed with altered treatment and demographics.

**Added value of this study**

We followed all persons in Sweden with incident epilepsy from 2006 to 2020 (n = 61,375) and compared survival in three incidence cohorts to age- and sex-matched controls until 2023 (median 4.5 years). In 2016–2020 compared to the earlier periods, newer ASMs had completely replaced older ones as first treatment. The adjusted risk of death was 1.99 that of controls in the first period and only slightly lower (hazard ratio [HR] 1.82 and 1.90 respectively) in the following periods. The HR for death in persons with cardiovascular disease was 1.59. A sensitivity analysis found a slight decrease in cardiovascular death between our cohorts. The study updates our understanding of mortality in epilepsy. It also shows that despite a shift in ASM therapy, mortality is still substantially elevated.

**Implications of all the available evidence**

Mortality for persons with epilepsy is substantial and newer ASMs do not alone seem to reduce the excess risk. The relative risk of cardiovascular death has decreased slightly, perhaps by use of newer ASMs, but a mortality gap remains. Tailored efforts are needed–focusing on seizures as well as comorbidities. Better integration of epilepsy care provision with other medical fields seems essential.

## Introduction

Epilepsy affects at least 50 million worldwide and is a prominent cause of mortality due to neurological disease.^1^^,^^2^ The Global Burden of Disease project found that non-structural epilepsy alone accounted for 5% of all disability-adjusted life years lost in neurology,^1^ notably this was exclusive of secondary epilepsies. In countries with ageing populations, secondary epilepsies due to vascular disease or neurodegenerative disorders become more prevalent.^3^ Although comorbidities make understanding of epilepsy mortality more challenging in older persons, studies including all epilepsy and are needed for adequate health care policy. The bidirectional association between late-onset epilepsy and vascular risk is becoming increasingly clear; midlife vascular risk factors affect epilepsy risk, and late-onset seizures can herald vascular events.^4^^,^^5^ Similar associations exist for neurodegenerative disorders; biochemical indicators of amyloid pathology in midlife indicate a higher epilepsy risk.^6^

Mortality in epilepsy reflects seizure-related deaths as well as deaths associated with underlying etiologies or comorbidities such as stroke. The excess mortality includes Sudden Unexpected Death in Epilepsy (SUDEP), which occurs in approximately 1 per 1000 patient years and is the most common cause of death in younger persons with epilepsy.^7^^,^^8^ In what the International League Against Epilepsy defines as idiopathic generalized epilepsies (genetic generalized epilepsies), the risk of death is approximately two times that of the general population,^1^^,^^9^ and in secondary epilepsies, the risk of death is about three-fold increased.^9^ Causes of death vary with etiology. In epilepsy after stroke, cardiovascular causes are more common than seizures,^10^^,^^11^ whereas the latter seem to account for a significant proportion of deaths in posttraumatic epilepsy.^12^

A large Danish study found unchanged mortality in epilepsy between 2010-2015.^13^ Since then, two major shifts in epilepsy treatment have a potential impact on survival. Firstly, antiseizure medications (ASMs) with CYP450-enzyme-inducing properties have been largely replaced by non-inducing ASMs, some of which are associated with a lower risk of vascular events and better survival.^10^^,^^14^ Secondly, the increased awareness of the teratogenic effects of valproic acid has reduced use of this drug among women with epilepsy.^15^ Valproic acid is more efficacious than other medications with regard to seizure freedom in idiopathic generalized epilepsy,^16^ so reduced use has potential consequences for the occurrence of tonic-clonic seizures, a major SUDEP risk factor. Taken together, the developments in ASM selection may have divergent effects on survival for different patient groups with epilepsy; vascular risk may be of particular concern in those middle aged or with existing comorbidities and reduced use of valproate may have a greater influence on childhood onset or idiopathic generalized epilepsies.

Studies on mortality in epilepsy face several methodological difficulties,^1^^,^^17^ including ascertainment of epilepsy and follow-up. The ideal method seems to be incident cohorts; prevalence cohorts aggregate more severe epilepsy.^9^^,^^17^ We therefore used comprehensive Swedish registers to study mortality in incidence cohorts with epilepsy onset in 2006–2023. We adhered to the ILAE terminology with regards to epilepsy classification and included all epilepsy; both idiopathic generalized and acquired forms. Our objective was to determine if there had been shifts in ASM prescription patterns and changes in epilepsy mortality in all incident patients 2006–2010, 2011–2015, and 2016–2020, and four subgroups that could be influenced by altered ASM habits: epilepsy onset >50 years of age, patients with cardiovascular comorbidities, idiopathic generalized epilepsy, or epilepsy onset <20 years of age.

## Methods

### Study population

The cohort was identified using the Swedish Health Registers, specifically the National Patient Register (NPR) and the National Prescribed Drug Register (DR). These registers contain data, including diagnostic codes, from inpatient care, outpatient specialist visits, and emergency room visits, and all prescription drug dispensations at pharmacies in Sweden. Reporting to the NPR is mandatory for all health care providers and pharmacies. Persons with epilepsy were identified by The National Board of Health and Welfare, the government register holder, and for each case, Statistics Sweden, a government agency, identified three comparators from the general population, matched for age and sex at the time of the epilepsy diagnosis. We then used the following inclusion criteria: first diagnosis of epilepsy (ICD-10: G40) after 1 Jan 2006 and before the 31 December 2020 in the NPR as a primary or secondary diagnosis, and at least one dispensation of an ASM (ATC code N03A, not gabapentin or pregabalin) after the epilepsy diagnosis (n = 377,988 epilepsy and comparators). The combination of an epilepsy diagnosis and ASM dispensation increases the specificity for identifying epilepsy in administrative data.^18^ The NPR has all data from specialized outpatient care since 2001 and all inpatient care since 1987, this allowed at least five years for identification of prevalent epilepsy, in agreement with the ILAE epidemiology commission report.^19^ The comparators formed reference cohorts for each period epilepsy cohorts, but were not individually matched to cases in paired analyses (see below). We excluded individuals with inconsistent data regarding death dates or a re-used personal identification number (n = 239) Persons with epilepsy were excluded if they: (1) retrieved ASMs more than three months before the first seizure-related diagnosis (G40, G41, R56.8, n = 17,864); (2) had any seizure-related diagnosis or ASM retrieval before January 1, 2006 or first ASM after 31 December, 2020 (n = 14,524); or (3) only used ASMs before epilepsy or did not retrieve ASMs (n = 575), or (4) had all comparators excluded (n = 19). The reason for inclusion of other codes than G40 in exclusion criteria was that the one-seizure ILAE definition of epilepsy was introduced only in 2014,^20^ so prevalent cases before that may only be captured by codes for single seizure (R56.8), status epilepticus (G41), as well as epilepsy (G40). Comparators were excluded if they had any seizure-related diagnosis (n = 3024), had been treated with ASMs (n = 3889), died before the ASM start of their matched person with epilepsy (n = 522) or whose epilepsy case was excluded (n = 99,309).

### Outcomes

Date of death was obtained from the Swedish Cause of Death Register, which has complete coverage. To avoid immortal time bias due to the dual requirement of an epilepsy diagnosis and an ASM prescription, patients with epilepsy were followed from the first use of ASM following the index diagnosis date (baseline) until death, emigration (1.6% in the epilepsy group and 1.8% in the comparator group), or the end of the follow-up period for each time frame—whichever occurred first. Patients were followed until three years after the last inclusion date for each period; December 31, 2013, for the period 2006–2010; December 31, 2018, for the period 2011–2015; and December 31, 2023, for the period 2016–2020. This three year period after each period was defined by the latest available register data (2023).

### Demographics and comorbidities

Information on age, sex, and comorbidities (binary) at the index date (first ASM dispensation) were obtained from the NPR. We focused on comorbidities that increase the risk of epilepsy, since survival and etiology have been shown to be linked in epilepsy.^9^ Stroke was defined as ICD-10: I60–I64; TBI as ICD-10: S06, S020, S021 S027, S029; dementia as ICD-10: F00–F03, G30, G31; brain tumour as ICD-10: C71, C793, D430, D32, D330; brain infection as ICD-10: A84, B004, A390, G00, G01, A17, A066, B431, G060, G079, A85, A86, B011, B020, B050, B262, B602, A87, B003, B010, B051, B261, B021, B375, B384, B582, G02; diabetes as ICD-10: E10, E11; cardiovascular diseases as ICD-10: I00–I99 (not I10, not I60-64, not I11.9-I16); depression or anxiety as ICD-10: F3, F4; intellectual disability ICD-10: F70-79, congenital disorder (yes, no) as Q00-Q07, Q90-Q99, cancer as ICD-10 C. Although not causal with regards to death, intellectual disability and congenital disorders were used as surrogate markers intended to adjust for any developments in care of patient groups with various congenital syndromes over the long study period. We calculated Charlson Comorbidity Index using an adaptation previously described for Swedish registers.^21^

### Statistical analyses

Continuous variables were described by mean, standard deviation (SD), median and range, and categorical variables by frequency and percentage. For statistical comparisons between two groups Fisher’s exact test was used for binary variables, and t-test for continuous variables. Cox regression was used to estimate the excess risk of dying among persons with epilepsy compared to comparators in each calendar period (main analysis). The matching was intentionally disregarded to enable more flexible adjustment and better control of potential confounders in the analysis. Crude event rates were calculated as number of events divided by follow-up time and expressed by 100 person-years. The 95% confidence interval (CI) were estimated by using exact Poisson limits. To test the difference between the calendar periods an interaction term between the period and group (epilepsy versus comparator) was included in the model. Unadjusted (matched groups for age and sex), and adjusted analyses for age, sex and for comorbidities as listed above, were performed overall and for all subgroups except for the generalized epilepsy due to limited number of individuals with an event in the comparator group. In the analysis of risk factors, Cox regression was used to evaluate the association between comorbidities at baseline and mortality in epilepsy cases, separately per each calendar period and evaluating the interaction of variable importance over time. Additionally a linear year-by-year trend was studied for 5-year mortality outcome. In a separate sensitivity analysis, only death with certain registered causes was counted as an outcome; cardiovascular or non-infectious death, defined by ICD-10 codes in the Cause of Death Register as underlying cause I00–I99 or absence of A00-A99, B00–B99 or U00–U99 as underlying or contributing cause, respectively. Because the Covid-19 pandemic influenced the last incidence cohort, we also analyzed cardiovascular death and non-infectious death in a separate analysis, without adjustment for cardiovascular morbidity in the first analysis. All tests were two-tailed. Alpha was set at 0.001 to define significant results in the main analysis, and 0.05 in other exploratory analyses. Missing month and day for diagnosis, medication or death was substituted with July 1 for the relevant year. In case of missing a specific diagnosis, medication or death date, the day of the month was set to 15. In total, 772 (0.3%) dates among epilepsy individuals required imputation, and 478 (0.3%) among comparators. Otherwise, missing data were not an issue in this study. The variables are based on the National Patient Register and the Prescribed Drug Register. If a diagnosis or medication is recorded in the respective register, it is classified as an event, otherwise, it is treated as no event.The statistical analyses were performed by a statistical contractor, working under accreditation. All analyses were performed using SAS software version 9.4 (SAS Institute Inc., Cary, NC, USA).

### Subgroups

In addition to the overall population, we categorized patients into four subgroups; age >50 at index, persons with cardiovascular disease (including stroke) at index, persons with generalized epilepsy, and age >20 at index. Cardiovascular disease including stroke was defined as defined as a preexisting ICD-10: I00–I99 (not I10, not I11.9-I16) in the in- and out-patient register as main or secondary diagnosis. Generalized epilepsy (referring to ILAE idiopathic/genetic generalized epilepsies) was defined as ICD G40.3, G40.4, G40.7 and age of onset <35 years. In all subgroups, all analyses were performed for the overall group and stratified by sex.

### Ethical permission and data handling

The Ethics Review Authority approved the study (approval number 2023–5598) and waived the need for informed consent. All data were cross-referenced by the register holders using the personal identification number unique to inhabitants of Sweden, and anonymized before we were given access to them.

### Role of the funding source

The funding sources had no role in study design; in the collection, analysis, and interpretation of data; in the writing of the report; and in the decision to submit the paper for publication.

## Results

### Epilepsy demographics

The three incident epilepsy cohorts in 2006–2010 (n = 18,497), 2011–2015 (n = 21,329), and 2016–2020 (n = 21,549) had no major differences in proportion of males/females and prevalence of comorbidities (Table 1). The proportion of persons with epilepsy onset after 50 accounted for >50% throughout our studied period. Among patients >50 the mean age at first epilepsy treatment increased from 72 years in the first period to 73 years (Supplementary Table S1), a similar difference was seen in patients with cardiovascular disease including stroke (Supplementary Table S1). Demographic and characteristics for persons with generalized epilepsy and persons <20 years of age were largely unchanged between the study periods (Supplementary Table S1).

**Table 1:** The three incident cohorts with demographic and clinical characteristics.

	2006–2010 N = 71,579		2011–2015 N = 82,621		2016–2020 N = 83,823	
	Epilepsy N = 18,497	Comparators N = 53,082	Epilepsy N = 21,329	Comparators N = 61,292	Epilepsy N = 21,549	Comparators N = 62,274
Age at inclusion mean (SD)	47.4 ± 28.7	47.3 ± 28.8	47.3 ± 29.2	47.1 ± 29.3	46.2 ± 30.0	45.8 ± 30.0
Age at inclusion						
≤50	8821 (47.7%)	25,442 (47.9%)	10,239 (48.0%)	29,632 (48.3%)	10,666 (49.5%)	31,090 (49.9%)
>50	9676 (52.3%)	27,640 (52.1%)	11,090 (52.0%)	31,660 (51.7%)	10,883 (50.5%)	31,184 (50.1%)
Sex						
Male	9619 (52.0%)	27,696 (52.2%)	11,402 (53.5%)	32,792 (53.5%)	11,754 (54.5%)	34,034 (54.7%)
Female	8878 (48.0%)	25,386 (47.8%)	9927 (46.5%)	28,500 (46.5%)	9795 (45.5%)	28,240 (45.3%)
Stroke	4468 (24.2%)	1650 (3.1%)	5107 (23.9%)	2081 (3.4%)	4781 (22.2%)	2084 (3.3%)
Traumatic brain injury	980 (5.3%)	329 (0.6%)	1179 (5.5%)	461 (0.8%)	1138 (5.3%)	434 (0.7%)
Dementia	965 (5.2%)	716 (1.3%)	1298 (6.1%)	976 (1.6%)	1171 (5.4%)	907 (1.5%)
Brain tumour	1542 (8.3%)	77 (0.1%)	1732 (8.1%)	127 (0.2%)	1720 (8.0%)	147 (0.2%)
Brain infection	264 (1.4%)	128 (0.2%)	368 (1.7%)	203 (0.3%)	322 (1.5%)	230 (0.4%)
Diabetes	1518 (8.2%)	1663 (3.1%)	2151 (10.1%)	2231 (3.6%)	2111 (9.8%)	2309 (3.7%)
Cardiovascular disease	5831 (31.5%)	5161 (9.7%)	7129 (33.4%)	6825 (11.1%)	6814 (31.6%)	6682 (10.7%)
Depression or anxiety	1472 (8.0%)	883 (1.7%)	2009 (9.4%)	1311 (2.1%)	1900 (8.8%)	1461 (2.3%)
Intellectual disability	467 (2.5%)	65 (0.1%)	636 (3.0%)	134 (0.2%)	768 (3.6%)	181 (0.3%)
Congenital disorder	339 (1.8%)	51 (0.1%)	509 (2.4%)	59 (0.1%)	565 (2.6%)	80 (0.1%)
Cancer	1982 (10.7%)	2298 (4.3%)	2333 (10.9%)	2970 (4.8%)	2276 (10.6%)	3131 (5.0%)
Charlson index	1.1 ± 1.6 0.0 (0.0–14.0)	0.3 ± 0.8 0.0 (0.0–10.0)	1.2 ± 1.8 0.0 (0.0–13.0)	0.3 ± 0.9 0.0 (0.0–13.0)	1.2 ± 1.8 0.0 (0.0–13.0)	0.3 ± 1.0 0.0 (0.0–13.0) n = 62,274

Mean (SD), median (minimum-maximum) presented for continuous variables and frequency and percentage for categorical variables.

### Epilepsy treatment

**Fig. 1:**
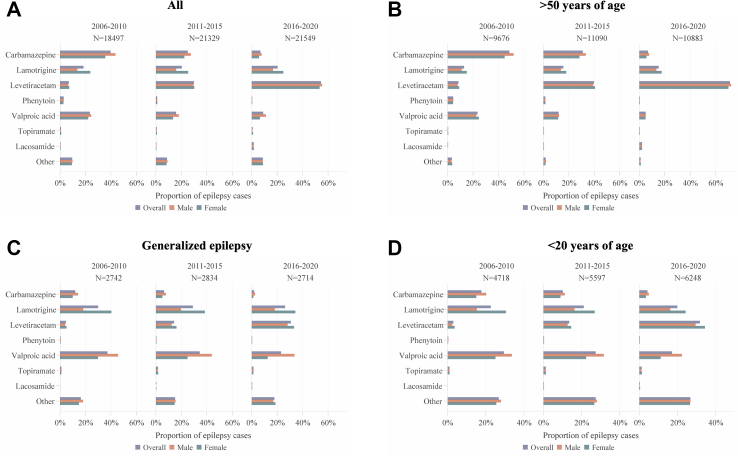
First antiseizure medication in the studied time periods overall (A) and in the subgroups; persons with incident epilepsy >50 years of age (B), generalized epilepsy (C), and <20 years of age (D).

The first ASMs shifted markedly over the study time (Fig. 1). In the total population carbamazepine and valproic acid were the most common choices in 2006–2010, but these had been replaced by levetiracetam in 2016–2020. The shift from carbamazepine to levetiracetam was most evident in older patients. Valproate became less common in generalized epilepsy, particularly in women.

### Mortality – all patients

**Fig. 2:**
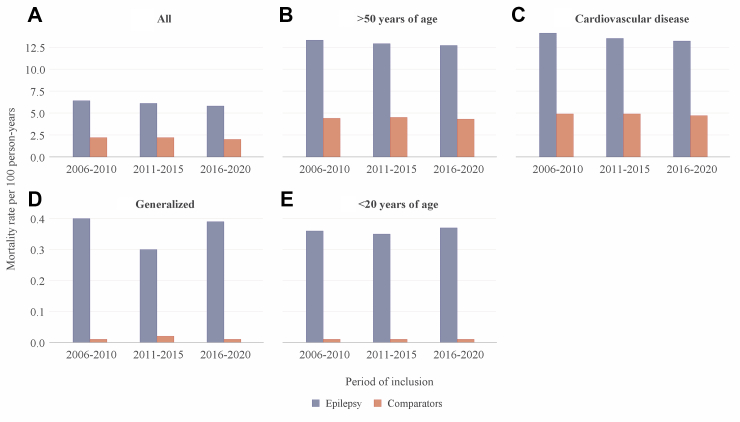
Unadjusted mortality rates in the three time periods for all patients (A), persons with incident epilepsy >50 years of age (B), cardiovascular disease (C), generalized epilepsy (D), and <20 years of age (E).

**Fig. 3:**
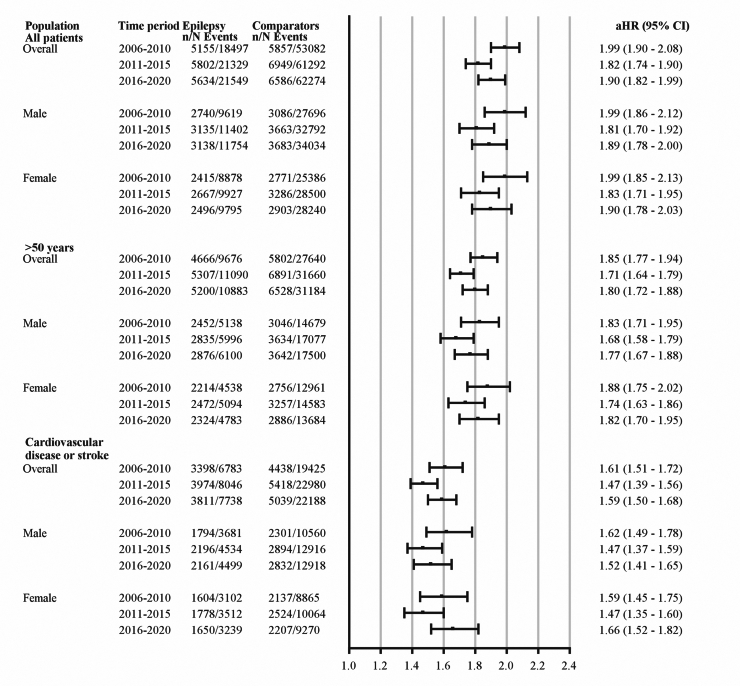
Adjusted hazard ratio of death for cases versus comparators, overall and per sex in all patients, patients >50, and patients with cardiovascular disease.

The unadjusted mortality rates (Fig. 2) for a median of 4.5 (IQR 3.2–6.0) years follow-up after index diagnosis in 2006–2010 was 6.4 (95% CI 6.2–6.6) per 100 person years compared to 2.2 (95% CI 2.1–2.3) for comparators, resulting in an unadjusted hazard ratio (HR) of 2.88 (95% CI 2.78–2.99) in 2006–2010. For 2016–2020, the event rates for cases and comparators were 5.8 (95% CI 5.6–5.9) and 2.0 (95% CI 2.0–2.1) per 100 person years, respectively, during the median follow-up of 4.7 (IQR 3.3–6.3) years, and the unadjusted HR for death 2.78 (95% CI 2.69–2.88). The difference in HR between the time periods was not significant (Supplementary Table S2). We also performed a year-by-year analysis of 5-year mortality that did not show a significant linear trend for differences in mortality (p = 0.09). Per sex, the unadjusted HR for death in males was 2.93 (2.78–3.09) for 2006–2010 and 2.78 (2.65–2.92) in 2016–2020. The unadjusted HR for death in females was 2.83 (2.68–2.99) in 2006–2010 and 2.79 (2.64–2.94) in 2016–2020. When the analysis was adjusted for age, sex and comorbidities, the excess risk of death explained by epilepsy versus comparators was lower in epilepsy incident in 2016–2020 (1.90, 95% CI 1.82–1.99) compared to 2006–2010 (1.99, 95% CI 1.90–2.08). The trend was consistent in sex-stratified analysis (Fig. 3).

### Subgroups

For patients >50 (Fig. 3, Supplementary Fig. S2), the adjusted HR of death compared to comparators was 1.85 (95% CI: 1.77–1.94) for the 2006–2010 cohort and 1.80 (95% CI: 1.72–1.88) for the 2016–2020 cohort. The adjusted HR for death in persons with cardiovascular disease (Fig. 3, Supplementary Fig. S3) was 1.61 (95% CI: 1.51–1.72) for persons with incident epilepsy in 2006–2010 and 1.59 (95% CI: 1.50–1.68) for 2016–2020. In persons with generalized epilepsy (Supplementary Fig. S4), the HR of death was not different between the time periods; 38.8 (95% CI:14.1–106.8) in 2006–2010 and 43.4 (95% CI: 15.8–119.6) in 2016–2020. A similar magnitude of excess risk was seen for persons with epilepsy incidence before the age of 20 (Supplementary Fig. S5). Because of the low number of deaths, adjustments were not possible in the young age group and in persons with generalized epilepsy.

### Cardiovascular and non-infectious death

In the subgroups over 50 years of age and cardiovascular disease, the HR of death seemed to have improved from 2006–2010 to 2011–2015, but then deteriorated again for the last period cohort 2016–2020, with follow-up until 2023. Event rates for cases were gradually lower (13.3-12.9-12.7 per in cases versus 4.4-4.5-4.3 in comparators per 100 person years) between all cohorts. In a separate analysis of only cardiovascular or non-infectious causes of death (Supplementary Tables S3 and S4, respectively), the adjusted HR for cardiovascular death for persons with epilepsy compared to comparators decreased from 2.0 (95% CI 1.86–2.15) in 2006–2010 to 1.73 (95% CI 1.60–1.87) in 2016–2020. There was no significant difference in the adjusted HR for non-infectious death (2.00, 95% CI: 1.91–2.10 in 2006–2010 versus 1.94, 95% CI: 1.85–2.03) in 2016–2020).

### Risk factors for death

We finally identified risk factors of death among persons with epilepsy during our three incident periods. Higher age at the start of ASM following the epilepsy diagnosis, or comorbidities before the start of ASM like stroke, dementia, brain tumor, diabetes, cardiovascular disease, cancer, and a high Charlson comorbidity index were associated with an increased risk of death in all periods (Table 2). Intellectual disability showed lower risk for death in all three calendar periods. Some of these risk factors became slightly stronger risk factors for death in the later time periods; the HR for death in persons with traumatic brain injury increased from HR 1.10 (95% CI: 0.98–1.24) in 2006–2010 to HR 1.42 (95% CI: 1.28–1.57) in 2016–2020. The HR for death with dementia increased from HR 4.61 (95% CI: 4.26–4.98) for epilepsy cases incident in 2006–2010 to 5.35 (95% CI: 4.98–5.75) in 2016–2020, and that of cardiovascular disease from HR 3.87 (95% CI: 3.66–4.09) for 2006–2010 to 4.37 (95% CI: 4.14–4.61) in 2016–2020.

**Table 2:** Univariable analysis of risk factors for death among patients with index epilepsy during 2006–2010, 2011–2015, and 2016–2020, during follow-up until three years after end of each period (median 4.5 years).

Variable	Value	2006–2010 N = 18,497		2011–2015 N = 21,329		2016–2020 N = 21,549	
		Mortality rate per 100 patient-years (95% CI)	HR (95% CI)	Mortality rate per 100 patient-years (95% CI)	HR (95% CI)	Mortality rate per 100 patient-years (95% CI)	HR (95% CI)
Sex (Male versus Female)	Male	6.6 (6.3–6.8)	1.06 (1.00–1.11)	6.2 (6.0–6.4)	1.02 (0.97–1.08)	5.9 (5.7–6.1)	1.05 (1.00–1.11)
	Female	6.2 (6.0–6.5)		6.0 (5.8–6.3)		5.6 (5.4–5.8)	
Age at inclusion (Per 1 year increase)	≥median	13.6 (13.2–14.0)	1.05 (1.05–1.06)	13.2 (12.9–13.6)	1.06 (1.05–1.06)	12.7 (12.4–13.1)	1.06 (1.05–1.06)
	<median	1.2 (1.1–1.3)		1.0 (0.9–1.1)		0.8 (0.7–0.8)	
Stroke (Yes versus No)	Yes	14.0 (13.5–14.6)	2.94 (2.78–3.11)	13.6 (13.1–14.2)	3.02 (2.87–3.18)	13.3 (12.8–13.8)	3.13 (2.96–3.30)
	No	4.5 (4.3–4.7)		4.3 (4.1–4.4)		4.1 (3.9–4.2)	
TBI (Yes versus No)	Yes	7.0 (6.3–7.9)	1.10 (0.98–1.24)	7.4 (6.7–8.2)	1.22 (1.10–1.35)	8.1 (7.3–8.9)	1.42 (1.28–1.57)
	No	6.4 (6.2–6.5)		6.0 (5.9–6.2)		5.6 (5.5–5.8)	
Dementia (Yes versus No)	Yes	30.0 (27.9–32.2)	4.61 (4.26–4.98)	30.1 (28.2–32.0)	4.95 (4.62–5.30)	30.6 (28.6–32.7)	5.35 (4.98–5.75)
	No	5.7 (5.5–5.8)		5.2 (5.1–5.4)		5.0 (4.8–5.1)	
Brain tumour (Yes versus No)	Yes	23.8 (22.3–25.3)	3.93 (3.66–4.22)	21.7 (20.4–23.1)	3.75 (3.51–4.01)	23.0 (21.6–24.4)	4.31 (4.03–4.60)
	No	5.5 (5.3–5.6)		5.3 (5.1–5.4)		4.9 (4.7–5.0)	
Brain infection (Yes versus No)	Yes	5.4 (4.2–6.9)	0.85 (0.66–1.09)	5.3 (4.3–6.5)	0.87 (0.71–1.07)	5.8 (4.6–7.1)	1.01 (0.82–1.25)
	No	6.4 (6.2–6.6)		6.1 (6.0–6.3)		5.8 (5.6–5.9)	
Diabetes (Yes versus No)	Yes	15.7 (14.7–16.8)	2.55 (2.37–2.75)	15.3 (14.4–16.2)	2.69 (2.52–2.87)	15.6 (14.7–16.6)	2.96 (2.77–3.16)
	No	5.8 (5.6–5.9)		5.3 (5.2–5.5)		5.0 (4.8–5.1)	
Cardiovascular diseases (Yes versus No)	Yes	14.8 (14.2–15.3)	3.87 (3.66–4.09)	14.4 (14.0–14.9)	4.43 (4.20–4.67)	13.8 (13.4–14.3)	4.37 (4.14–4.61)
	No	3.6 (3.4–3.7)		3.1 (2.9–3.2)		3.0 (2.9–3.1)	
Depression or anxiety (Yes versus No)	Yes	6.4 (5.8–7.0)	0.99 (0.89–1.09)	6.5 (6.0–7.1)	1.07 (0.98–1.17)	6.0 (5.4–6.5)	1.04 (0.95–1.14)
	No	6.4 (6.2–6.6)		6.1 (5.9–6.2)		5.7 (5.6–5.9)	
Intellectual disability (Yes versus No)	Yes	1.7 (1.2–2.3)	0.26 (0.19–0.36)	1.9 (1.5–2.5)	0.31 (0.24–0.40)	1.7 (1.3–2.1)	0.29 (0.23–0.37)
	No	6.5 (6.4–6.7)		6.3 (6.1–6.4)		5.9 (5.8–6.1)	
Inborn disorder (Yes versus No)	Yes	4.3 (3.3–5.5)	0.67 (0.52–0.85)	4.6 (3.8–5.5)	0.75 (0.62–0.90)	4.6 (3.8–5.5)	0.80 (0.67–0.96)
	No	6.4 (6.3–6.6)		6.2 (6.0–6.3)		5.8 (5.6–5.9)	
Cancer (Yes versus No)	Yes	28.8 (27.3–30.4)	5.11 (4.80–5.43)	26.8 (25.5–28.1)	5.05 (4.76–5.35)	26.8 (25.5–28.2)	5.51 (5.20–5.84)
	No	5.0 (4.8–5.1)		4.7 (4.6–4.9)		4.4 (4.3–4.5)	
Charlson index (Per 1 unit increase)	≥1	13.9 (13.5–14.3)	1.52 (1.50–1.53)	13.2 (12.8–13.5)	1.44 (1.43–1.45)	12.6 (12.2–13.0)	1.45 (1.44–1.46)
	0	1.7 (1.5–1.8)		1.4 (1.3–1.5)		1.4 (1.3–1.5)	

## Discussion

We provide a comprehensive analysis of mortality and risk factors for death in epilepsy, using a register-based approach with minimal loss-to follow-up, given the mandatory reporting to the registers used. Our results supplement the Global Burden of Disease study, by including also secondary epilepsies and taking the full range of comorbidities into account. Our main finding is that despite improved pharmacotherapy, mortality in epilepsy remains high. In younger patients the risk of death is 40 times that of age-matched comparators. SUDEP accounts for at least 20% of all death in persons with epilepsy aged 16–50 and 36% of all deaths in those under 16 in Sweden,^7^ and our findings call for raised ambitions regarding particularly freedom from tonic-clonic seizures (the main SUDEP risk factor) in epilepsy management. In patients over the age of 50, cardiovascular disease and other comorbidities are increasingly important risk factors for death. This finding calls for increased awareness of late-onset epilepsy as an indicator of vascular risk and better integration of epilepsy care with other medical areas.

The overall risk of death associated with epilepsy compared to age- and sex-matched comparators was approximately 2.8, which is towards the higher end of standardized mortality ratios found in high income countries in previous studies.^17^^,^^22^^,^^23^ It is slightly higher than previous and older estimates from Sweden^24^ suggesting that there may be an actual increase in the risk of death associated with epilepsy as the population ages. This would agree with European calculations in the Global Burden of Disease study 2017, according to which deaths in epilepsy in Europe had increased by 53% from 1990 to 2017.^25^ Our adjusted analyses indicated that the excess risk of death associated with epilepsy when comorbidities are taken into account was about two-fold increased for the entire population, and 1.5 times increased for persons with cardiovascular disease. Interestingly, the death rates in all our subgroups, including the youngest and those with generalized epilepsy, exceeded the risk of SUDEP (about 0.1/100 patient years),^7^ which suggests that seizure prevention alone will not suffice to reduce all excess mortality.

Changes in epilepsy pharmacotherapy with non-enzyme inducing ASMs replacing older drugs has been implemented in Sweden and we detected a profound shift in the first ASM used in persons with incident epilepsy in 2016–2020 compared to 2006–2010; the non-enzyme inducer levetiracetam now dominates. The overall increased risk of death associated with epilepsy decreased only slightly between our time periods and remained clearly elevated above that of age- and sex-matched comparators. This extends the timeline and conclusion from a previous Danish study, which also assessed all epilepsy and found largely unchanged mortality between 2010 and 2015^13^; before the shift to non-enzyme inducing ASMs in our study. These results seems puzzling, given that the benefits of newer generation ASMs with regards to vascular risk have been demonstrated both epidemiologically and mechanistically, at least for persons with established cardiovascular risk,^10^^,^^26^ which constituted a third of our cohort. In the subgroup with cardiovascular disease, we did not find a substantial decrease in mortality as the ASM habits changed, but importantly the excess risk of death was only 1.6, which is lower compared to epilepsy overall. It is possible that newer ASMs were particularly used in persons with cardiovascular comorbidities already in the first period, and that some of the benefits of the newer ASMs therefore predate our investigation. Our sensitivity analysis did find a significant decrease in the risk of specifically cardiovascular death, and the less detrimental effects of the new ASMs on vascular health could be a contributor. If so, further interventions are needed to fully close the remaining mortality gap. The newer ASMs allow better cardiovascular prognosis, but higher ambitions are also needed regarding the actual cardiovascular interventions, like better management of blood pressure, blood lipids, etc.

A previous period study on a hospital-based cohort in Austria showed that relative risk of death had decreased substantially in incident epilepsy from 3.0 in the 1980s to 1.4 in 2007.^27^ Such a shift is not detectable on a whole-country level in Sweden. For younger patients, there has been no marked improvement in epilepsy mortality over our study period. In 2019, Sweden introduced national guidelines for epilepsy care that pointed out severe gaps in equal access to care and too low ambitions with regards to epilepsy management. The national guidelines were in place only for the last years of our study period, which were also influenced by the pandemic. Our findings of the very high excess risk support the continued implementation of the national guidelines and raised ambitions in epilepsy care. This is particularly important given reported associations between a lower risk of death in epilepsy and access to specialized epilepsy care.^28^ Other important areas of study are socioeconomic determinants of premature mortality in epilepsy.^17^

Methodologically, studies on mortality in epilepsy pose several challenges. We used the most robust epilepsy definition for administrative data and incidence cohorts, which has been a recommended approach for adequate assessment of mortality.^17^ In addition, comprehensive Swedish national registers ensures equal detection of mortality in cases and comparators throughout our study period. We adjusted for comorbidities that are common epilepsy etiologies, since survival in acquired epilepsy is often linked to etiology.^9^ We also adjusted for a comorbidity index. Nonetheless, these are relatively crude markers and residual confounding is a limitation. We aimed to study incident cases based on appearance of diagnostic codes in registers, but some prevalent cases never treated in specialized care or that have remained undiagnosed until the start of our study periods may have escaped exclusion. Detection of comorbidities should be similar across epilepsy cases diagnosed in our three time periods, but because the used registers are limited to hospital-based care it is possible that detection of comorbidities treated only in primary care have been slightly less robust in comparators and that co-morbidities are more often diagnosed or registered in persons with epilepsy when they are treated for epilepsy. For this reason, we assessed risk factors of death in cases with epilepsy only. We also assessed only the first ASM, and future more detailed analysis of actual time on different ASMs in mono- and polytherapy may be more revealing. We hypothesize that non-enzyme inducing ASMs may be involved in the slightly reduced mortality seen in persons with epilepsy and cardiovascular disease, but firm conclusions will require further study. Emigration was not accounted for apart from censoring, but this was relatively minor. We did not include persons with single seizures in the comparator groups, which may have been artificially healthy, although the effect is likely minor. The last of our final cohort with epilepsy onset in 2016–2020 will have had their follow-up during the pandemic, when health services were not operating optimally. The increased relative risk of death in the last period cohort fits well with our overall interpretation that non-epilepsy health issues are important.

Our results call for greater efforts in reducing excess mortality in epilepsy. The findings regarding comorbidities being important risk factors for death suggest that in the increasing proportion of middle aged and older patients, new-onset epilepsy should be seen as much as a warning with regard to brain health as a seizure problem. Vascular and neurodegenerative comorbidities merit increased attention in epilepsy care.

We conclude that over the last two decades, mortality in epilepsy has remained largely unchanged relative to age- and sex matched comparators, despite increased use of non-inducing ASMs. There has possibly been a decrease in the risk of cardiovascular death, but this does not seem sufficient to reduce all excess mortality. Given the heterogeneity of epilepsy, efforts should be tailored to the specific challenges for different patient groups and could include access to specialized epilepsy care as well as better focus on comorbidities.

## Contributors

Johan Zelano: conceptualization, study design, data collection, data interpretation, writing, supervision. André Idegård: data collection, data interpretation, revision. David Larsson: study design, data interpretation, revision.

## Data sharing statement

The underlying data is available from Swedish national registers upon request, but cannot be shared by the authors because of confidentially agreements with the register holders and Swedish privacy regulations.

## Declaration of interests

JZ declares being a speaker at non-branded educational events organized by UCB, Eisai, Orion pharma, and Angelini pharma (honoraria), royalty for neurology textbooks from Studentlitteratur AB, Liber AB, and CRC Press, and being investigator in clinical trials sponsored by Bial, UCB, SK life science, GW Pharma, and Angilini Pharma as an employee of Sahlgrenska University Hospital (no personal compensation). Outside of the submitted work, he has received research grants in the last 36 months from his employers and these non-profit entities: Linnea and Josef Carlsson foundation, Edit Jacobsson donationsfond, John and Brit Wennerström foundation for neurological research, Promobilia foundation, Biegler foundation, Margaretahemmet foundation. DL and AI have no disclosures.
