# Supplementary material for: Trends in epilepsy mortality of three incidence cohorts across 2006–2023 in Sweden: a matched register-based study
Source: Lancet Reg Health Eur. 2025 Jul 23;56:101388. doi: 10.1016/j.lanepe.2025.101388 (PMC12310390; doi:10.1016/j.lanepe.2025.101388)

Suppl figure 1
All patients

Population	Time period	Epilepsy n/N Events	Comparators n/N Events	HR (95% CI)
				**Unadjusted**
Overall	2006-2010	5155/18497	5857/53082	2.88 (2.78 - 2.99)
	2011-2015	5802/21329	6949/61292	2.73 (2.63 - 2.82)
	2016-2020	5634/21549	6586/62274	2.78 (2.69 - 2.88)
Male	2006-2010	2740/9619	3086/27696	2.93 (2.78 - 3.09)
	2011-2015	3135/11402	3663/32792	2.80 (2.67 - 2.94)
	2016-2020	3138/11754	3683/34034	2.78 (2.65 - 2.92)
Female	2006-2010	2415/8878	2771/25386	2.83 (2.68 - 2.99)
	2011-2015	2667/9927	3286/28500	2.65 (2.51 - 2.78)
	2016-2020	2496/9795	2903/28240	2.79 (2.64 - 2.94)
				**Adjusted**
Overall	2006-2010	5155/18497	5857/53082	1.99 (1.90 - 2.08)
	2011-2015	5802/21329	6949/61292	1.82 (1.74 - 1.90)
	2016-2020	5634/21549	6586/62274	1.90 (1.82 - 1.99)
Male	2006-2010	2740/9619	3086/27696	1.99 (1.86 - 2.12)
	2011-2015	3135/11402	3663/32792	1.81 (1.70 - 1.92)
	2016-2020	3138/11754	3683/34034	1.89 (1.78 - 2.00)
Female	2006-2010	2415/8878	2771/25386	1.99 (1.85 - 2.13)
	2011-2015	2667/9927	3286/28500	1.83 (1.71 - 1.95)
	2016-2020	2496/9795	2903/28240	1.90 (1.78 - 2.03)

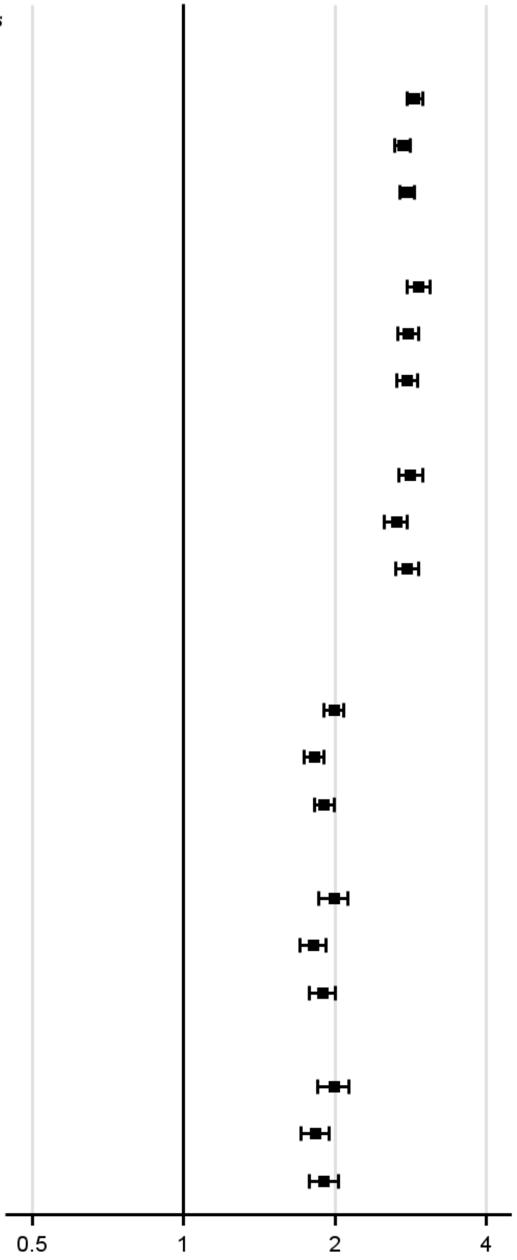

Suppl figure 2
Incident epilepsy over 50

Population	Time period	Epilepsy n/N Events	Comparators n/N Events	HR (95% CI)
				**Unadjusted**
Overall	2006-2010	4666/9676	5802/27640	2.97 (2.85 - 3.08)
	2011-2015	5307/11090	6891/31660	2.83 (2.73 - 2.93)
	2016-2020	5200/10883	6528/31184	2.92 (2.81 - 3.02)
Male	2006-2010	2452/5138	3046/14679	2.96 (2.81 - 3.12)
	2011-2015	2835/5996	3634/17077	2.84 (2.70 - 2.98)
	2016-2020	2876/6100	3642/17500	2.87 (2.73 - 3.02)
Female	2006-2010	2214/4538	2756/12961	2.97 (2.81 - 3.14)
	2011-2015	2472/5094	3257/14583	2.81 (2.67 - 2.96)
	2016-2020	2324/4783	2886/13684	2.98 (2.82 - 3.14)
				**Adjusted**
Overall	2006-2010	4666/9676	5802/27640	1.85 (1.77 - 1.94)
	2011-2015	5307/11090	6891/31660	1.71 (1.64 - 1.79)
	2016-2020	5200/10883	6528/31184	1.80 (1.72 - 1.88)
Male	2006-2010	2452/5138	3046/14679	1.83 (1.71 - 1.95)
	2011-2015	2835/5996	3634/17077	1.68 (1.58 - 1.79)
	2016-2020	2876/6100	3642/17500	1.77 (1.67 - 1.88)
Female	2006-2010	2214/4538	2756/12961	1.88 (1.75 - 2.02)
	2011-2015	2472/5094	3257/14583	1.74 (1.63 - 1.86)
	2016-2020	2324/4783	2886/13684	1.82 (1.70 - 1.95)

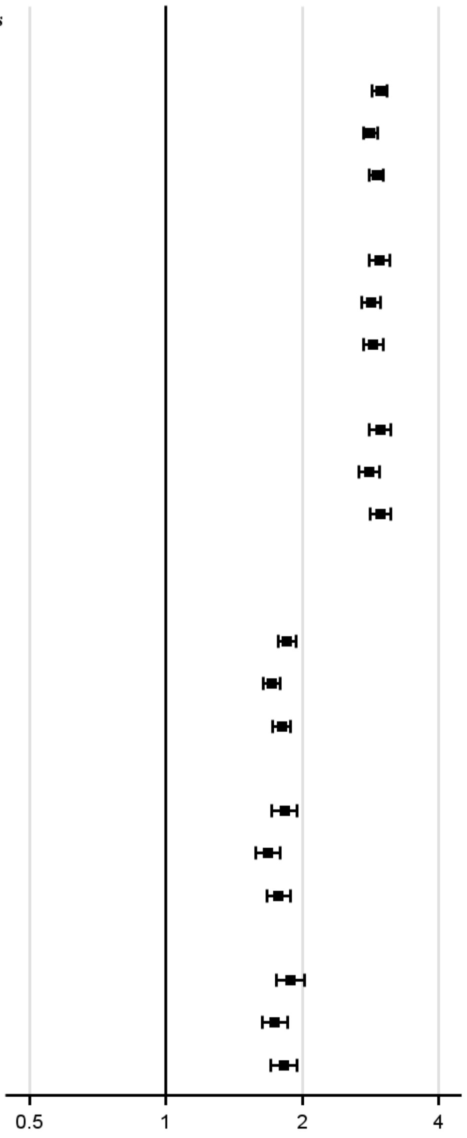

Suppl figure 3 Cardiovascular disease including stroke

Population	Time period	Epilepsy n/N Events	Comparators n/N Events	HR (95% CI)
				**Unadjusted**
Overall	2006-2010	3398/6783	4438/19425	2.84 (2.71 - 2.97)
	2011-2015	3974/8046	5418/22980	2.69 (2.58 - 2.80)
	2016-2020	3811/7738	5039/22188	2.77 (2.66 - 2.89)
Male	2006-2010	1794/3681	2301/10560	2.87 (2.70 - 3.06)
	2011-2015	2196/4534	2894/12916	2.75 (2.60 - 2.91)
	2016-2020	2161/4499	2832/12918	2.77 (2.62 - 2.93)
Female	2006-2010	1604/3102	2137/8865	2.80 (2.62 - 2.99)
	2011-2015	1778/3512	2524/10064	2.63 (2.47 - 2.79)
	2016-2020	1650/3239	2207/9270	2.77 (2.60 - 2.95)
				**Adjusted**
Overall	2006-2010	3398/6783	4438/19425	1.61 (1.51 - 1.72)
	2011-2015	3974/8046	5418/22980	1.47 (1.39 - 1.56)
	2016-2020	3811/7738	5039/22188	1.59 (1.50 - 1.68)
Male	2006-2010	1794/3681	2301/10560	1.62 (1.49 - 1.78)
	2011-2015	2196/4534	2894/12916	1.47 (1.37 - 1.59)
	2016-2020	2161/4499	2832/12918	1.52 (1.41 - 1.65)
Female	2006-2010	1604/3102	2137/8865	1.59 (1.45 - 1.75)
	2011-2015	1778/3512	2524/10064	1.47 (1.35 - 1.60)
	2016-2020	1650/3239	2207/9270	1.66 (1.52 - 1.82)

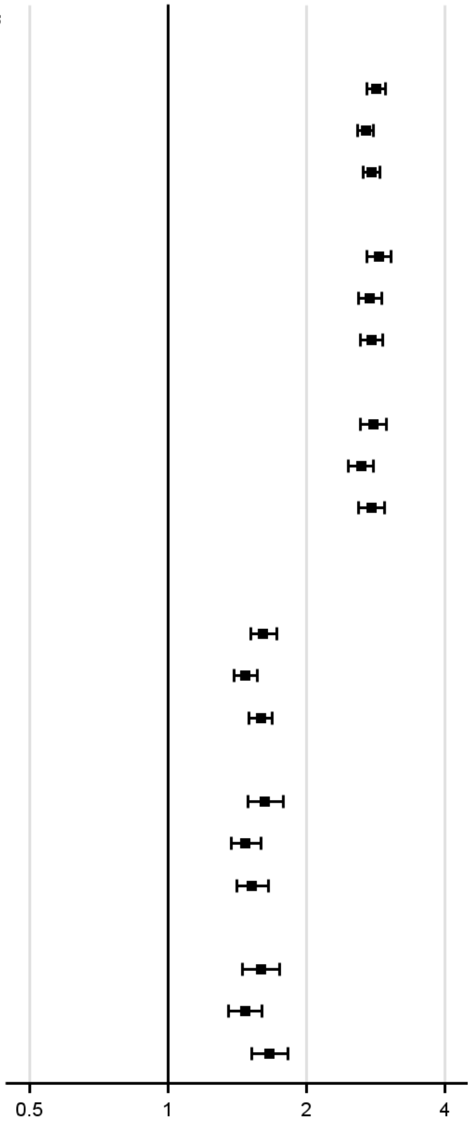

Suppl figure 4
Generalized epilepsy

Population	Time period	Epilepsy n/N Events	Comparators n/N Events	HR (95% CI) Unadjusted
Overall	2006-2010	58/2742	4/7237	38.77 (14.08 - 106.75)
	2011-2015	46/2834	7/7595	17.80 (8.04 - 39.43)
	2016-2020	59/2714	4/7919	43.44 (15.78 - 119.58)
Male	2006-2010	35/1287	3/3409	31.46 (9.68 - 102.29)
	2011-2015	26/1427	5/3830	14.12 (5.42 - 36.76)
	2016-2020	41/1350	2/3934	60.50 (14.63 - 250.14)
Female	2006-2010	23/1455	1/3828	61.04 (8.24 - 451.98)
	2011-2015	20/1407	2/3765	27.02 (6.32 - 115.61)
	2016-2020	18/1364	2/3985	26.48 (6.14 - 114.10)

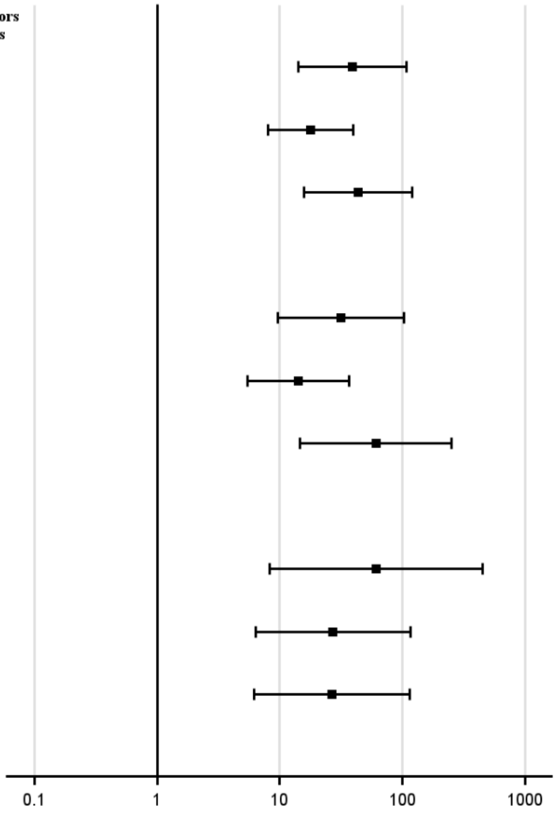

Suppl figure 5
Age < 20 no brain tumor

Population	Time period	Epilepsy n/N Events	Comparators n/N Events	HR (95% CI) Unadjusted
Overall	2006-2010	90/4718	5/13627	52.25 (21.24 - 128.55)
	2011-2015	104/5597	10/16308	30.56 (15.97 - 58.48)
	2016-2020	125/6248	12/18334	30.85 (17.06 - 55.79)
Male	2006-2010	52/2436	2/7095	76.35 (18.60 - 313.45)
	2011-2015	60/3024	7/8841	25.29 (11.56 - 55.33)
	2016-2020	73/3367	8/9900	27.12 (13.07 - 56.28)
Female	2006-2010	38/2282	3/6532	36.38 (11.23 - 117.84)
	2011-2015	44/2573	3/7467	42.88 (13.32 - 138.09)
	2016-2020	52/2881	4/8434	38.35 (13.87 - 106.03)

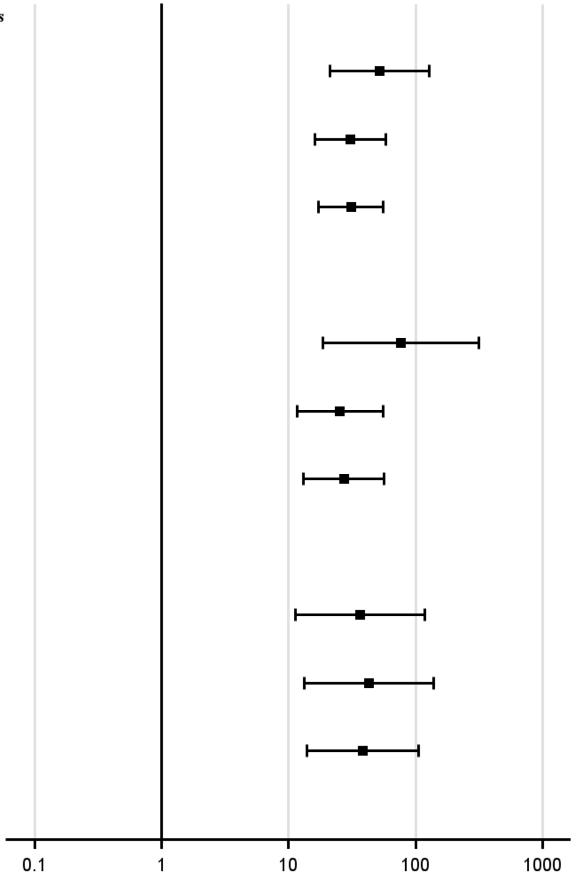

Supplement: Supplementary Figs. S1–S5 [file mmc1.pdf]
