# Supplementary material for: Trends in epilepsy mortality of three incidence cohorts across 2006–2023 in Sweden: a matched register-based study
Source: Lancet Reg Health Eur. 2025 Jul 23;56:101388. doi: 10.1016/j.lanepe.2025.101388 (PMC12310390; doi:10.1016/j.lanepe.2025.101388)
Supplement: Supplementary Tables S1–S4 [file mmc2.docx]

**Suppl Table 1 Demographics and clinical characteristics of the subgroups**

| **Subgroup**  **>50 years** | **2006-2010 N=37316** | | **2011-2015 N=42750** | | | **2016-2020 N=42067** | | |
| --- | --- | --- | --- | --- | --- | --- | --- | --- |
| **Variable** | **Epilepsy N=9676** | **Comparators N=27640** | **Epilepsy N=11090** | **Comparators N=31660** | | **Epilepsy N=10883** | **Comparators N=31184** | |
| Age at inclusion (mean, SD) | 71.8±11.1 n=9676 | 71.8±11.1  n=27640 | 72.5±11.0  n=11090 | 72.5±11.0  n=31660 | | 73.1±10.8  n=10883 | 73.1±10.9  n=31184 | |
| Sex |  |  |  |  | |  |  | |
| Male | 5138 (53.1%) | 14679 (53.1%) | 5996 (54.1%) | 17077 (53.9%) | | 6100 (56.1%) | 17500 (56.1%) | |
| Female | 4538 (46.9%) | 12961 (46.9%) | 5094 (45.9%) | 14583 (46.1%) | | 4783 (43.9%) | 13684 (43.9%) | |
| Stroke | 4082 (42.2%) | 1630 (5.9%) | 4677 (42.2%) | 2069 (6.5%) | | 4404 (40.5%) | 2067 (6.6%) | |
| TBI | 591 (6.1%) | 153 (0.6%) | 780 (7.0%) | 250 (0.8%) | | 755 (6.9%) | 222 (0.7%) | |
| Dementia | 938 (9.7%) | 715 (2.6%) | 1272 (11.5%) | 974 (3.1%) | | 1143 (10.5%) | 905 (2.9%) | |
| Brain tumour | 1101 (11.4%) | 67 (0.2%) | 1247 (11.2%) | 112 (0.4%) | | 1309 (12.0%) | 124 (0.4%) | |
| Brain infection | 136 (1.4%) | 56 (0.2%) | 206 (1.9%) | 89 (0.3%) | | 202 (1.9%) | 111 (0.4%) | |
| Diabetes | 1375 (14.2%) | 1520 (5.5%) | 1940 (17.5%) | 2051 (6.5%) | | 1946 (17.9%) | 2116 (6.8%) | |
| Cardiovascular diseases | 5266 (54.4%) | 4917 (17.8%) | 6465 (58.3%) | 6476 (20.5%) | | 6249 (57.4%) | 6386 (20.5%) | |
| Depression or anxiety | 803 (8.3%) | 527 (1.9%) | 1041 (9.4%) | 725 (2.3%) | | 953 (8.8%) | 652 (2.1%) | |
| Intellectual disability | 35 (0.4%) | 11 (0.0%) | 71 (0.6%) | 28 (0.1%) | | 66 (0.6%) | 22 (0.1%) | |
| Congenital disorder | 49 (0.5%) | 4 (0.0%) | 103 (0.9%) | 10 (0.0%) | | 99 (0.9%) | 8 (0.0%) | |
| Cancer | 1640 (16.9%) | 2209 (8.0%) | 1965 (17.7%) | 2872 (9.1%) | | 1987 (18.3%) | 3045 (9.8%) | |
| Charlson index | 1.7±1.8 1.0 (0.0 - 14.0) n=9676 | 0.5±1.1 0.0 (0.0 - 10.0) n=27640 | 2.0±2.0 1.0 (0.0 - 13.0) n=11090 | 0.6±1.2 0.0 (0.0 - 13.0) n=31660 | | 2.1±2.1 1.0 (0.0 - 13.0) n=10883 | 0.6±1.3 0.0 (0.0 - 13.0) n=31184 | |
| **Subgroup**  **Cardiovascular disease** | **2006-2010 N=26208** | | **2011-2015 N=31026** | | | **2016-2020 N=29926** | | |
| **Variable** | **Epilepsy N=6783** | **Comparators N=19425** | **Epilepsy N=8046** | **Comparators N=22980** | | **Epilepsy N=7738** | **Comparators N=22188** | |
| Age at inclusion (mean, SD) | 70.1±17.0 n=6783 | 70.0±17.0  n=19425 | 70.6±17.1  n=8046 | 70.5±17.1  n=22980 | | 71.2±17.4  n=7738 | 71.1±17.5  n=22188 | |
| Age at inclusion |  |  |  |  | |  |  | |
| ≤50 | 730 (10.8%) | 2097 (10.8%) | 837 (10.4%) | 2411 (10.5%) | | 728 (9.4%) | 2105 (9.5%) | |
| >50 | 6053 (89.2%) | 17328 (89.2%) | 7209 (89.6%) | 20569 (89.5%) | | 7010 (90.6%) | 20083 (90.5%) | |
| Sex |  |  |  |  | |  |  | |
| Male | 3681 (54.3%) | 10560 (54.4%) | 4534 (56.4%) | 12916 (56.2%) | | 4499 (58.1%) | 12918 (58.2%) | |
| Female | 3102 (45.7%) | 8865 (45.6%) | 3512 (43.6%) | 10064 (43.8%) | | 3239 (41.9%) | 9270 (41.8%) | |
| Stroke | 4468 (65.9%) | 1219 (6.3%) | 5107 (63.5%) | 1553 (6.8%) | | 4781 (61.8%) | 1521 (6.9%) | |
| TBI | 478 (7.0%) | 118 (0.6%) | 679 (8.4%) | 187 (0.8%) | | 662 (8.6%) | 187 (0.8%) | |
| Dementia | 638 (9.4%) | 552 (2.8%) | 851 (10.6%) | 774 (3.4%) | | 746 (9.6%) | 710 (3.2%) | |
| Brain tumour | 421 (6.2%) | 49 (0.3%) | 549 (6.8%) | 69 (0.3%) | | 602 (7.8%) | 96 (0.4%) | |
| Brain infection | 100 (1.5%) | 49 (0.3%) | 161 (2.0%) | 72 (0.3%) | | 145 (1.9%) | 72 (0.3%) | |
| Diabetes | 1121 (16.5%) | 1068 (5.5%) | 1640 (20.4%) | 1480 (6.4%) | | 1596 (20.6%) | 1492 (6.7%) | |
| Cardiovascular diseases | 5831 (86.0%) | 3533 (18.2%) | 7129 (88.6%) | 4789 (20.8%) | | 6814 (88.1%) | 4634 (20.9%) | |
| Depression or anxiety | 603 (8.9%) | 367 (1.9%) | 815 (10.1%) | 506 (2.2%) | | 767 (9.9%) | 462 (2.1%) | |
| Intellectual disability | 35 (0.5%) | 8 (0.0%) | 67 (0.8%) | 24 (0.1%) | | 57 (0.7%) | 19 (0.1%) | |
| Congenital disorder | 39 (0.6%) | 8 (0.0%) | 74 (0.9%) | 7 (0.0%) | | 64 (0.8%) | 11 (0.0%) | |
| Cancer | 955 (14.1%) | 1546 (8.0%) | 1241 (15.4%) | 2098 (9.1%) | | 1279 (16.5%) | 2156 (9.7%) | |
| Charlson index | 2.1±1.7 2.0 (0.0 - 14.0) n=6783 | 0.5±1.1 0.0 (0.0 - 10.0) n=19425 | 2.4±1.9 2.0 (0.0 - 13.0) n=8046 | 0.6±1.2 0.0 (0.0 - 13.0) n=22980 | | 2.5±2.0 2.0 (0.0 - 13.0) n=7738 | 0.6±1.3 0.0 (0.0 - 13.0) n=22188 | |
| **Subgroup**  **Generalized epilepsy** | **2006-2010 N=9979** | | **2011-2015 N=10429** | | | **2016-2020 N=10633** | | |
| **Variable** | **Epilepsy N=2742** | **Comparators N=7237** | **Epilepsy N=2834** | **Comparators N=7595** | | **Epilepsy N=2714** | **Comparators N=7919** | |
| Age at inclusion | 11.7±8.4  n=2742 | 11.7±8.5  n=7237 | 11.8±8.3  n=2834 | 11.7±8.4  n=7595 | | 11.7±8.3  n=2714 | 11.7±8.3  n=7919 | |
| Sex |  |  |  |  | |  |  | |
| Male | 1287 (46.9%) | 3409 (47.1%) | 1427 (50.4%) | 3830 (50.4%) | | 1350 (49.7%) | 3934 (49.7%) | |
| Female | 1455 (53.1%) | 3828 (52.9%) | 1407 (49.6%) | 3765 (49.6%) | | 1364 (50.3%) | 3985 (50.3%) | |
| Stroke | 25 (0.9%) | 1 (0.0%) | 28 (1.0%) | 0 (0.0%) | | 26 (1.0%) | 4 (0.1%) | |
| TBI | 87 (3.2%) | 63 (0.9%) | 75 (2.6%) | 65 (0.9%) | | 80 (2.9%) | 65 (0.8%) | |
| Dementia | 7 (0.3%) | 0 (0.0%) | 4 (0.1%) | 0 (0.0%) | | 5 (0.2%) | 0 (0.0%) | |
| Brain tumour | 24 (0.9%) | 1 (0.0%) | 36 (1.3%) | 4 (0.1%) | | 8 (0.3%) | 4 (0.1%) | |
| Brain infection | 25 (0.9%) | 15 (0.2%) | 24 (0.8%) | 31 (0.4%) | | 10 (0.4%) | 21 (0.3%) | |
| Diabetes | 21 (0.8%) | 20 (0.3%) | 42 (1.5%) | 26 (0.3%) | | 26 (1.0%) | 29 (0.4%) | |
| Cardiovascular diseases | 60 (2.2%) | 31 (0.4%) | 65 (2.3%) | 41 (0.5%) | | 59 (2.2%) | 53 (0.7%) | |
| Depression or anxiety | 98 (3.6%) | 58 (0.8%) | 138 (4.9%) | 118 (1.6%) | | 136 (5.0%) | 187 (2.4%) | |
| Intellectual disability | 147 (5.4%) | 17 (0.2%) | 156 (5.5%) | 30 (0.4%) | | 151 (5.6%) | 32 (0.4%) | |
| Congenital disorder | 116 (4.2%) | 13 (0.2%) | 124 (4.4%) | 19 (0.3%) | | 129 (4.8%) | 21 (0.3%) | |
| Cancer | 25 (0.9%) | 12 (0.2%) | 27 (1.0%) | 12 (0.2%) | | 8 (0.3%) | 7 (0.1%) | |
| Charlson index | 0.2±0.5 0.0 (0.0 - 3.0) n=2742 | 0.0±0.2 0.0 (0.0 - 6.0) n=7237 | 0.2±0.6 0.0 (0.0 - 8.0) n=2834 | 0.0±0.2 0.0 (0.0 - 6.0) n=7595 | | 0.2±0.6 0.0 (0.0 - 9.0) n=2714 | 0.0±0.2 0.0 (0.0 - 4.0) n=7919 | |
| **Subgroup**  **Age <20 years+no tumor** | **2006-2010 N=18345** | | **2011-2015 N=21905** | | **2016-2020 N=24582** | | |  |
| **Variable** | **Epilepsy N=4718** | **Comparators N=13627** | **Epilepsy N=5597** | **Comparators N=16308** | | **Epilepsy N=6248** | **Comparators N=18334** | |
| Age at inclusion | 8.6±5.9  n=4718 | 8.6±5.9  n=13627 | 8.7±5.7 n=5597 | 8.6±5.7  n=16308 | | 8.6±5.6  n=6248 | 8.6±5.6 8n=18334 | |
| Sex |  |  |  |  | |  |  | |
| Male | 2436 (51.6%) | 7095 (52.1%) | 3024 (54.0%) | 8841 (54.2%) | | 3367 (53.9%) | 9900 (54.0%) | |
| Female | 2282 (48.4%) | 6532 (47.9%) | 2573 (46.0%) | 7467 (45.8%) | | 2881 (46.1%) | 8434 (46.0%) | |
| Stroke | 56 (1.2%) | 3 (0.0%) | 74 (1.3%) | 1 (0.0%) | | 86 (1.4%) | 7 (0.0%) | |
| TBI | 133 (2.8%) | 120 (0.9%) | 130 (2.3%) | 118 (0.7%) | | 138 (2.2%) | 129 (0.7%) | |
| Dementia | 9 (0.2%) | 0 (0.0%) | 10 (0.2%) | 0 (0.0%) | | 10 (0.2%) | 0 (0.0%) | |
| Brain tumour | 3 (0.1%) | 2 (0.0%) | 9 (0.2%) | 4 (0.0%) | | 4 (0.1%) | 10 (0.1%) | |
| Brain infection | 64 (1.4%) | 38 (0.3%) | 58 (1.0%) | 58 (0.4%) | | 50 (0.8%) | 50 (0.3%) | |
| Diabetes | 27 (0.6%) | 44 (0.3%) | 49 (0.9%) | 49 (0.3%) | | 46 (0.7%) | 72 (0.4%) | |
| Cardiovascular diseases | 95 (2.0%) | 49 (0.4%) | 121 (2.2%) | 77 (0.5%) | | 130 (2.1%) | 94 (0.5%) | |
| Depression or anxiety | 96 (2.0%) | 59 (0.4%) | 141 (2.5%) | 140 (0.9%) | | 196 (3.1%) | 256 (1.4%) | |
| Intellectual disability | 332 (7.0%) | 35 (0.3%) | 395 (7.1%) | 55 (0.3%) | | 497 (8.0%) | 84 (0.5%) | |
| Congenital disorder | 233 (4.9%) | 37 (0.3%) | 300 (5.4%) | 40 (0.2%) | | 374 (6.0%) | 50 (0.3%) | |
| Cancer | 16 (0.3%) | 11 (0.1%) | 20 (0.4%) | 13 (0.1%) | | 11 (0.2%) | 27 (0.1%) | |
| Charlson index | 0.2±0.6 0.0 (0.0 - 6.0) n=4718 | 0.0±0.2 0.0 (0.0 - 6.0) n=13627 | 0.2±0.6 0.0 (0.0 - 7.0) n=5597 | 0.1±0.2 0.0 (0.0 - 7.0) n=16308 | | 0.2±0.6 0.0 (0.0 - 9.0) n=6248 | 0.1±0.3 0.0 (0.0 - 6.0) n=18334 | |

Supplemental Table 2 - Cox regression of HR for death in epilepsy versus reference group (comparators).

|  | | | **Epilepsy** | | | **Comparators** | | | **Epilepsy vs. Comparators** | | **Interaction** | |
| --- | --- | --- | --- | --- | --- | --- | --- | --- | --- | --- | --- | --- |
| **Subgroup** | **Period** | **Period** | **n/N (%) events No. events/ Follow-up time** | **Follow-up time Median (IQR)** | **Event rate (95% CI) per 100 person- years** | **n/N (%) events No. events/ Follow-up time** | **Follow-up time Median (IQR)** | **Event rate (95% CI) per 100 person- years** | **Hazard ratio (95% CI) p-value** | *** Hazard ratio (95% CI) p-value** | **p-value** | *** p-value** |
| ***Overall - population*** |  |  |  |  |  |  |  |  |  |  |  |  |
|  | **2006-2010 vs. 2016-2020** | **2006-2010** | 5155/18497 (27.9%) | 4.50 (3.20-6.02) Sum=80503 | 6.4 (6.2-6.6) | 5857/53082 (11.0%) | 5.03 (3.82-6.36) Sum=266935 | 2.2 (2.1-2.3) | 2.88 (2.78 - 2.99) p=.0001 | 1.99 (1.90 - 2.08) p<.0001 |  |  |
|  |  | **2016-2020** | 5634/21549 (26.1%) | 4.68 (3.28-6.27) Sum=97904 | 5.8 (5.6-5.9) | 6586/62274 (10.6%) | 5.21 (3.92-6.59) Sum=321887 | 2.0 (2.0-2.1) | 2.78 (2.69 - 2.88) p<.0001 | 1.90 (1.82 - 1.99) p<.0001 | 0.17 | <.0001 |
|  | **2006-2010 vs. 2011-2015** | **2006-2010** | 5155/18497 (27.9%) | 4.50 (3.20-6.02) Sum=80503 | 6.4 (6.2-6.6) | 5857/53082 (11.0%) | 5.03 (3.82-6.36) Sum=266935 | 2.2 (2.1-2.3) | 2.88 (2.78 - 2.99) p=.0001 | 1.99 (1.90 - 2.08) p<.0001 |  |  |
|  |  | **2011-2015** | 5802/21329 (27.2%) | 4.60 (3.22-6.17) Sum=94850 | 6.1 (6.0-6.3) | 6949/61292 (11.3%) | 5.14 (3.88-6.53) Sum=313595 | 2.2 (2.2-2.3) | 2.73 (2.63 - 2.82) p<.0001 | 1.82 (1.74 - 1.90) p<.0001 | 0.033 | <.0001 |
|  | **2011-2015 vs. 2016-2020** | **2011-2015** | 5802/21329 (27.2%) | 4.60 (3.22-6.17) Sum=94850 | 6.1 (6.0-6.3) | 6949/61292 (11.3%) | 5.14 (3.88-6.53) Sum=313595 | 2.2 (2.2-2.3) | 2.73 (2.63 - 2.82) p<.0001 | 1.82 (1.74 - 1.90) p<.0001 |  |  |
|  |  | **2016-2020** | 5634/21549 (26.1%) | 4.68 (3.28-6.27) Sum=97904 | 5.8 (5.6-5.9) | 6586/62274 (10.6%) | 5.21 (3.92-6.59) Sum=321887 | 2.0 (2.0-2.1) | 2.78 (2.69 - 2.88) p<.0001 | 1.90 (1.82 - 1.99) p<.0001 | 0.44 | 0.88 |
| ***Male - population*** |  |  |  |  |  |  |  |  |  |  |  |  |
|  | **2006-2010 vs. 2016-2020** | **2006-2010** | 2740/9619 (28.5%) | 4.49 (3.17-6.01) Sum=41660 | 6.6 (6.3-6.8) | 3086/27696 (11.1%) | 5.03 (3.83-6.34) Sum=139306 | 2.2 (2.1-2.3) | 2.93 (2.78 - 3.09) p<.0001 | 1.99 (1.86 - 2.12) p<.0001 |  |  |
|  |  | **2016-2020** | 3138/11754 (26.7%) | 4.67 (3.26-6.24) Sum=53176 | 5.9 (5.7-6.1) | 3683/34034 (10.8%) | 5.20 (3.92-6.57) Sum=175377 | 2.1 (2.0-2.2) | 2.78 (2.65 - 2.92) p<.0001 | 1.89 (1.78 - 2.00) p<.0001 | 0.13 | <.0001 |
|  | **2006-2010 vs. 2011-2015** | **2006-2010** | 2740/9619 (28.5%) | 4.49 (3.17-6.01) Sum=41660 | 6.6 (6.3-6.8) | 3086/27696 (11.1%) | 5.03 (3.83-6.34) Sum=139306 | 2.2 (2.1-2.3) | 2.93 (2.78 - 3.09) p<.0001 | 1.99 (1.86 - 2.12) p<.0001 |  |  |
|  |  | **2011-2015** | 3135/11402 (27.5%) | 4.61 (3.22-6.17) Sum=50688 | 6.2 (6.0-6.4) | 3663/32792 (11.2%) | 5.16 (3.88-6.54) Sum=167932 | 2.2 (2.1-2.3) | 2.80 (2.67 - 2.94) p<.0001 | 1.81 (1.70 - 1.92) p<.0001 | 0.20 | 0.0009 |
|  | **2011-2015 vs. 2016-2020** | **2011-2015** | 3135/11402 (27.5%) | 4.61 (3.22-6.17) Sum=50688 | 6.2 (6.0-6.4) | 3663/32792 (11.2%) | 5.16 (3.88-6.54) Sum=167932 | 2.2 (2.1-2.3) | 2.80 (2.67 - 2.94) p<.0001 | 1.81 (1.70 - 1.92) p<.0001 |  |  |
|  |  | **2016-2020** | 3138/11754 (26.7%) | 4.67 (3.26-6.24) Sum=53176 | 5.9 (5.7-6.1) | 3683/34034 (10.8%) | 5.20 (3.92-6.57) Sum=175377 | 2.1 (2.0-2.2) | 2.78 (2.65 - 2.92) p<.0001 | 1.89 (1.78 - 2.00) p<.0001 | 0.81 | 0.39 |
| ***Female - population*** |  |  |  |  |  |  |  |  |  |  |  |  |
|  | **2006-2010 vs. 2016-2020** | **2006-2010** | 2415/8878 (27.2%) | 4.52 (3.21-6.03) Sum=38843 | 6.2 (6.0-6.5) | 2771/25386 (10.9%) | 5.03 (3.81-6.38) Sum=127629 | 2.2 (2.1-2.3) | 2.83 (2.68 - 2.99) p<.0001 | 1.99 (1.85 - 2.13) p<.0001 |  |  |
|  |  | **2016-2020** | 2496/9795 (25.5%) | 4.71 (3.31-6.30) Sum=44728 | 5.6 (5.4-5.8) | 2903/28240 (10.3%) | 5.22 (3.92-6.62) Sum=146511 | 2.0 (1.9-2.1) | 2.79 (2.64 - 2.94) p<.0001 | 1.90 (1.78 - 2.03) p<.0001 | 0.69 | 0.0050 |
|  | **2006-2010 vs. 2011-2015** | **2006-2010** | 2415/8878 (27.2%) | 4.52 (3.21-6.03) Sum=38843 | 6.2 (6.0-6.5) | 2771/25386 (10.9%) | 5.03 (3.81-6.38) Sum=127629 | 2.2 (2.1-2.3) | 2.83 (2.68 - 2.99) p<.0001 | 1.99 (1.85 - 2.13) p<.0001 |  |  |
|  |  | **2011-2015** | 2667/9927 (26.9%) | 4.58 (3.23-6.18) Sum=44162 | 6.0 (5.8-6.3) | 3286/28500 (11.5%) | 5.12 (3.88-6.52) Sum=145663 | 2.3 (2.2-2.3) | 2.65 (2.51 - 2.78) p<.0001 | 1.83 (1.71 - 1.95) p<.0001 | 0.08 | 0.0003 |
|  | **2011-2015 vs. 2016-2020** | **2011-2015** | 2667/9927 (26.9%) | 4.58 (3.23-6.18) Sum=44162 | 6.0 (5.8-6.3) | 3286/28500 (11.5%) | 5.12 (3.88-6.52) Sum=145663 | 2.3 (2.2-2.3) | 2.65 (2.51 - 2.78) p<.0001 | 1.83 (1.71 - 1.95) p<.0001 |  |  |
|  |  | **2016-2020** | 2496/9795 (25.5%) | 4.71 (3.31-6.30) Sum=44728 | 5.6 (5.4-5.8) | 2903/28240 (10.3%) | 5.22 (3.92-6.62) Sum=146511 | 2.0 (1.9-2.1) | 2.79 (2.64 - 2.94) p<.0001 | 1.90 (1.78 - 2.03) p<.0001 | 0.17 | 0.41 |

**Suppl table 3 – Cox regression evaluating the risk of cardiovascular death.**

|  | | | **Epilepsy** | | | **Comparators** | | | **Epilepsy vs. Comparators** | | **Interaction** | |
| --- | --- | --- | --- | --- | --- | --- | --- | --- | --- | --- | --- | --- |
| **Analysis** | **Period** | **Period** | **n/N (%) events No. events/ Follow-up time** | **Follow-up time Median (IQR)** | **Event rate (95% CI) per 100 person- years** | **n/N (%) events No. events/ Follow-up time** | **Follow-up time Median (IQR)** | **Event rate (95% CI) per 100 person- years** | **Hazard ratio (95% CI) p-value** | *** Hazard ratio (95% CI) p-value** | **p-value** | *** p-value** |
| ***Overall - population*** |  |  |  |  |  |  |  |  |  |  |  |  |
|  | **2006-2010 vs. 2016-2019** | **2006-2010** | 2040/18497 (11.0%) | 4.50 (3.20-6.02) Sum=80503 | 2.5 (2.4-2.6) | 2471/53082 (4.7%) | 5.03 (3.82-6.36) Sum=266935 | 0.9 (0.9-1.0) | 2.71 (2.56 - 2.87) p<.0001 | 2.00 (1.86 - 2.15) p<.0001 |  |  |
|  |  | **2016-2019** | 1628/21549 (7.6%) | 4.68 (3.28-6.27) Sum=97904 | 1.7 (1.6-1.7) | 2136/62274 (3.4%) | 5.21 (3.92-6.59) Sum=321887 | 0.7 (0.6-0.7) | 2.48 (2.33 - 2.65) p<.0001 | 1.73 (1.60 - 1.87) p<.0001 | 0.048 | <.0001 |
|  | **2006-2010 vs. 2011-2015** | **2006-2010** | 2040/18497 (11.0%) | 4.50 (3.20-6.02) Sum=80503 | 2.5 (2.4-2.6) | 2471/53082 (4.7%) | 5.03 (3.82-6.36) Sum=266935 | 0.9 (0.9-1.0) | 2.71 (2.56 - 2.87) p<.0001 | 2.00 (1.86 - 2.15) p<.0001 |  |  |
|  |  | **2011-2015** | 2059/21329 (9.7%) | 4.60 (3.22-6.17) Sum=94850 | 2.2 (2.1-2.3) | 2732/61292 (4.5%) | 5.14 (3.88-6.53) Sum=313595 | 0.9 (0.8-0.9) | 2.46 (2.33 - 2.61) p<.0001 | 1.73 (1.62 - 1.86) p<.0001 | 0.025 | <.0001 |
|  | **2011-2015 vs. 2016-2019** | **2011-2015** | 2059/21329 (9.7%) | 4.60 (3.22-6.17) Sum=94850 | 2.2 (2.1-2.3) | 2732/61292 (4.5%) | 5.14 (3.88-6.53) Sum=313595 | 0.9 (0.8-0.9) | 2.46 (2.33 - 2.61) p<.0001 | 1.73 (1.62 - 1.86) p<.0001 |  |  |
|  |  | **2016-2019** | 1628/21549 (7.6%) | 4.68 (3.28-6.27) Sum=97904 | 1.7 (1.6-1.7) | 2136/62274 (3.4%) | 5.21 (3.92-6.59) Sum=321887 | 0.7 (0.6-0.7) | 2.48 (2.33 - 2.65) p<.0001 | 1.73 (1.60 - 1.87) p<.0001 | 0.88 | 0.41 |
| ***Male - population*** |  |  |  |  |  |  |  |  |  |  |  |  |
|  | **2006-2010 vs. 2016-2019** | **2006-2010** | 1021/9619 (10.6%) | 4.49 (3.17-6.01) Sum=41660 | 2.5 (2.3-2.6) | 1296/27696 (4.7%) | 5.03 (3.83-6.34) Sum=139306 | 0.9 (0.9-1.0) | 2.61 (2.41 - 2.83) p<.0001 | 1.93 (1.75 - 2.13) p<.0001 |  |  |
|  |  | **2016-2019** | 887/11754 (7.5%) | 4.67 (3.26-6.24) Sum=53176 | 1.7 (1.6-1.8) | 1166/34034 (3.4%) | 5.20 (3.92-6.57) Sum=175377 | 0.7 (0.6-0.7) | 2.49 (2.28 - 2.72) p<.0001 | 1.67 (1.50 - 1.85) p<.0001 | 0.43 | 0.0029 |
|  | **2006-2010 vs. 2011-2015** | **2006-2010** | 1021/9619 (10.6%) | 4.49 (3.17-6.01) Sum=41660 | 2.5 (2.3-2.6) | 1296/27696 (4.7%) | 5.03 (3.83-6.34) Sum=139306 | 0.9 (0.9-1.0) | 2.61 (2.41 - 2.83) p<.0001 | 1.93 (1.75 - 2.13) p<.0001 |  |  |
|  |  | **2011-2015** | 1063/11402 (9.3%) | 4.61 (3.22-6.17) Sum=50688 | 2.1 (2.0-2.2) | 1411/32792 (4.3%) | 5.16 (3.88-6.54) Sum=167932 | 0.8 (0.8-0.9) | 2.47 (2.28 - 2.67) p<.0001 | 1.76 (1.60 - 1.93) p<.0001 | 0.36 | 0.016 |
|  | **2011-2015 vs. 2016-2019** | **2011-2015** | 1063/11402 (9.3%) | 4.61 (3.22-6.17) Sum=50688 | 2.1 (2.0-2.2) | 1411/32792 (4.3%) | 5.16 (3.88-6.54) Sum=167932 | 0.8 (0.8-0.9) | 2.47 (2.28 - 2.67) p<.0001 | 1.76 (1.60 - 1.93) p<.0001 |  |  |
|  |  | **2016-2019** | 887/11754 (7.5%) | 4.67 (3.26-6.24) Sum=53176 | 1.7 (1.6-1.8) | 1166/34034 (3.4%) | 5.20 (3.92-6.57) Sum=175377 | 0.7 (0.6-0.7) | 2.49 (2.28 - 2.72) p<.0001 | 1.67 (1.50 - 1.85) p<.0001 | 0.92 | 0.52 |
| ***Female - population*** |  |  |  |  |  |  |  |  |  |  |  |  |
|  | **2006-2010 vs. 2016-2019** | **2006-2010** | 1019/8878 (11.5%) | 4.52 (3.21-6.03) Sum=38843 | 2.6 (2.5-2.8) | 1175/25386 (4.6%) | 5.03 (3.81-6.38) Sum=127629 | 0.9 (0.9-1.0) | 2.82 (2.59 - 3.06) p<.0001 | 2.07 (1.87 - 2.30) p<.0001 |  |  |
|  |  | **2016-2019** | 741/9795 (7.6%) | 4.71 (3.31-6.30) Sum=44728 | 1.7 (1.5-1.8) | 970/28240 (3.4%) | 5.22 (3.92-6.62) Sum=146511 | 0.7 (0.6-0.7) | 2.48 (2.25 - 2.73) p<.0001 | 1.81 (1.61 - 2.03) p<.0001 | 0.046 | 0.0003 |
|  | **2006-2010 vs. 2011-2015** | **2006-2010** | 1019/8878 (11.5%) | 4.52 (3.21-6.03) Sum=38843 | 2.6 (2.5-2.8) | 1175/25386 (4.6%) | 5.03 (3.81-6.38) Sum=127629 | 0.9 (0.9-1.0) | 2.82 (2.59 - 3.06) p<.0001 | 2.07 (1.87 - 2.30) p<.0001 |  |  |
|  |  | **2011-2015** | 996/9927 (10.0%) | 4.58 (3.23-6.18) Sum=44162 | 2.3 (2.1-2.4) | 1321/28500 (4.6%) | 5.12 (3.88-6.52) Sum=145663 | 0.9 (0.9-1.0) | 2.46 (2.26 - 2.67) p<.0001 | 1.69 (1.53 - 1.87) p<.0001 | 0.023 | 0.0006 |
|  | **2011-2015 vs. 2016-2019** | **2011-2015** | 996/9927 (10.0%) | 4.58 (3.23-6.18) Sum=44162 | 2.3 (2.1-2.4) | 1321/28500 (4.6%) | 5.12 (3.88-6.52) Sum=145663 | 0.9 (0.9-1.0) | 2.46 (2.26 - 2.67) p<.0001 | 1.69 (1.53 - 1.87) p<.0001 |  |  |
|  |  | **2016-2019** | 741/9795 (7.6%) | 4.71 (3.31-6.30) Sum=44728 | 1.7 (1.5-1.8) | 970/28240 (3.4%) | 5.22 (3.92-6.62) Sum=146511 | 0.7 (0.6-0.7) | 2.48 (2.25 - 2.73) p<.0001 | 1.81 (1.61 - 2.03) p<.0001 | 0.91 | 0.58 |
| Confidence interval for unadjusted event rates per 100 person years are obtained from exact Poisson confidence limits. Cox regression was used for time to any event presented by HR. * Adjusted for Age at inclusion, Sex, Stroke, TBI, Dementia, Brain tumour, Brain infection, Diabetes, Depression or anxiety, Intellectual disability, Inborn disorder, Cancer, Charlson index. The p-value for interaction was calculated between study group and each pairwise time period. | | | | | | | | | | | | |

**Suppl table 4. Cox regression evaluating the HR of non-infectious death**

|  | | | **Epilepsy** | | | **Comparators** | | | **Epilepsy vs. Comparators** | | **Interaction** | |
| --- | --- | --- | --- | --- | --- | --- | --- | --- | --- | --- | --- | --- |
| **Analysis** | **Period** | **Period** | **n/N (%) events No. events/ Follow-up time** | **Follow-up time Median (IQR)** | **Event rate (95% CI) per 100 person- years** | **n/N (%) events No. events/ Follow-up time** | **Follow-up time Median (IQR)** | **Event rate (95% CI) per 100 person- years** | **Hazard ratio (95% CI) p-value** | *** Hazard ratio (95% CI) p-value** | **p-value** | *** p-value** |
| ***Overall - population*** |  |  |  |  |  |  |  |  |  |  |  |  |
|  | **2006-2010 vs. 2016-2020** | **2006-2010** | 4818/18497 (26.0%) | 4.50 (3.20-6.02) Sum=80503 | 6.0 (5.8-6.2) | 5428/53082 (10.2%) | 5.03 (3.82-6.36) Sum=266935 | 2.0 (2.0-2.1) | 2.91 (2.80 - 3.02) p<.0001 | 2.00 (1.91 - 2.10) p<.0001 |  |  |
|  |  | **2016-2020** | 5006/21549 (23.2%) | 4.68 (3.28-6.27) Sum=97904 | 5.1 (5.0-5.3) | 5751/62274 (9.2%) | 5.21 (3.92-6.59) Sum=321887 | 1.8 (1.7-1.8) | 2.83 (2.72 - 2.94) p<.0001 | 1.94 (1.85 - 2.03) p<.0001 | 0.33 | <.0001 |
|  | **2006-2010 vs. 2011-2015** | **2006-2010** | 4818/18497 (26.0%) | 4.50 (3.20-6.02) Sum=80503 | 6.0 (5.8-6.2) | 5428/53082 (10.2%) | 5.03 (3.82-6.36) Sum=266935 | 2.0 (2.0-2.1) | 2.91 (2.80 - 3.02) p<.0001 | 2.00 (1.91 - 2.10) p<.0001 |  |  |
|  |  | **2011-2015** | 5363/21329 (25.1%) | 4.60 (3.22-6.17) Sum=94850 | 5.7 (5.5-5.8) | 6385/61292 (10.4%) | 5.14 (3.88-6.53) Sum=313595 | 2.0 (2.0-2.1) | 2.74 (2.64 - 2.84) p<.0001 | 1.84 (1.76 - 1.92) p<.0001 | 0.032 | <.0001 |
|  | **2011-2015 vs. 2016-2020** | **2011-2015** | 5363/21329 (25.1%) | 4.60 (3.22-6.17) Sum=94850 | 5.7 (5.5-5.8) | 6385/61292 (10.4%) | 5.14 (3.88-6.53) Sum=313595 | 2.0 (2.0-2.1) | 2.74 (2.64 - 2.84) p<.0001 | 1.84 (1.76 - 1.92) p<.0001 |  |  |
|  |  | **2016-2020** | 5006/21549 (23.2%) | 4.68 (3.28-6.27) Sum=97904 | 5.1 (5.0-5.3) | 5751/62274 (9.2%) | 5.21 (3.92-6.59) Sum=321887 | 1.8 (1.7-1.8) | 2.83 (2.72 - 2.94) p<.0001 | 1.94 (1.85 - 2.03) p<.0001 | 0.24 | 0.78 |
| ***Male - population*** |  |  |  |  |  |  |  |  |  |  |  |  |
|  | **2006-2010 vs. 2016-2020** | **2006-2010** | 2556/9619 (26.6%) | 4.49 (3.17-6.01) Sum=41660 | 6.1 (5.9-6.4) | 2866/27696 (10.3%) | 5.03 (3.83-6.34) Sum=139306 | 2.1 (2.0-2.1) | 2.94 (2.79 - 3.10) p<.0001 | 1.98 (1.86 - 2.12) p<.0001 |  |  |
|  |  | **2016-2020** | 2764/11754 (23.5%) | 4.67 (3.26-6.24) Sum=53176 | 5.2 (5.0-5.4) | 3172/34034 (9.3%) | 5.20 (3.92-6.57) Sum=175377 | 1.8 (1.7-1.9) | 2.84 (2.70 - 2.99) p<.0001 | 1.93 (1.81 - 2.05) p<.0001 | 0.34 | 0.0009 |
|  | **2006-2010 vs. 2011-2015** | **2006-2010** | 2556/9619 (26.6%) | 4.49 (3.17-6.01) Sum=41660 | 6.1 (5.9-6.4) | 2866/27696 (10.3%) | 5.03 (3.83-6.34) Sum=139306 | 2.1 (2.0-2.1) | 2.94 (2.79 - 3.10) p<.0001 | 1.98 (1.86 - 2.12) p<.0001 |  |  |
|  |  | **2011-2015** | 2864/11402 (25.1%) | 4.61 (3.22-6.17) Sum=50688 | 5.7 (5.4-5.9) | 3347/32792 (10.2%) | 5.16 (3.88-6.54) Sum=167932 | 2.0 (1.9-2.1) | 2.80 (2.66 - 2.94) p<.0001 | 1.81 (1.70 - 1.92) p<.0001 | 0.18 | 0.0013 |
|  | **2011-2015 vs. 2016-2020** | **2011-2015** | 2864/11402 (25.1%) | 4.61 (3.22-6.17) Sum=50688 | 5.7 (5.4-5.9) | 3347/32792 (10.2%) | 5.16 (3.88-6.54) Sum=167932 | 2.0 (1.9-2.1) | 2.80 (2.66 - 2.94) p<.0001 | 1.81 (1.70 - 1.92) p<.0001 |  |  |
|  |  | **2016-2020** | 2764/11754 (23.5%) | 4.67 (3.26-6.24) Sum=53176 | 5.2 (5.0-5.4) | 3172/34034 (9.3%) | 5.20 (3.92-6.57) Sum=175377 | 1.8 (1.7-1.9) | 2.84 (2.70 - 2.99) p<.0001 | 1.93 (1.81 - 2.05) p<.0001 | 0.69 | 0.84 |
| ***Female - population*** |  |  |  |  |  |  |  |  |  |  |  |  |
|  | **2006-2010 vs. 2016-2020** | **2006-2010** | 2262/8878 (25.5%) | 4.52 (3.21-6.03) Sum=38843 | 5.8 (5.6-6.1) | 2562/25386 (10.1%) | 5.03 (3.81-6.38) Sum=127629 | 2.0 (1.9-2.1) | 2.86 (2.71 - 3.03) p<.0001 | 2.02 (1.88 - 2.17) p<.0001 |  |  |
|  |  | **2016-2020** | 2242/9795 (22.9%) | 4.71 (3.31-6.30) Sum=44728 | 5.0 (4.8-5.2) | 2579/28240 (9.1%) | 5.22 (3.92-6.62) Sum=146511 | 1.8 (1.7-1.8) | 2.82 (2.66 - 2.98) p<.0001 | 1.94 (1.81 - 2.08) p<.0001 | 0.67 | 0.0083 |
|  | **2006-2010 vs. 2011-2015** | **2006-2010** | 2262/8878 (25.5%) | 4.52 (3.21-6.03) Sum=38843 | 5.8 (5.6-6.1) | 2562/25386 (10.1%) | 5.03 (3.81-6.38) Sum=127629 | 2.0 (1.9-2.1) | 2.86 (2.71 - 3.03) p<.0001 | 2.02 (1.88 - 2.17) p<.0001 |  |  |
|  |  | **2011-2015** | 2499/9927 (25.2%) | 4.58 (3.23-6.18) Sum=44162 | 5.7 (5.4-5.9) | 3038/28500 (10.7%) | 5.12 (3.88-6.52) Sum=145663 | 2.1 (2.0-2.2) | 2.68 (2.54 - 2.83) p<.0001 | 1.86 (1.74 - 1.99) p<.0001 | 0.09 | 0.0007 |
|  | **2011-2015 vs. 2016-2020** | **2011-2015** | 2499/9927 (25.2%) | 4.58 (3.23-6.18) Sum=44162 | 5.7 (5.4-5.9) | 3038/28500 (10.7%) | 5.12 (3.88-6.52) Sum=145663 | 2.1 (2.0-2.2) | 2.68 (2.54 - 2.83) p<.0001 | 1.86 (1.74 - 1.99) p<.0001 |  |  |
|  |  | **2016-2020** | 2242/9795 (22.9%) | 4.71 (3.31-6.30) Sum=44728 | 5.0 (4.8-5.2) | 2579/28240 (9.1%) | 5.22 (3.92-6.62) Sum=146511 | 1.8 (1.7-1.8) | 2.82 (2.66 - 2.98) p<.0001 | 1.94 (1.81 - 2.08) p<.0001 | 0.21 | 0.47 |
| Confidence interval for unadjusted event rates per 100 person years are obtained from exact Poisson confidence limits. Cox regression was used for time to any event presented by HR. * Adjusted for Age at inclusion, Sex, Stroke, TBI, Dementia, Brain tumour, Brain infection, Diabetes, Cardiovascular diseases, Depression or anxiety, Intellectual disability, Congenital disorder, Cancer, Charlson index. The p-value for interaction was calculated between study group and each pairwise time period. The event is non-infection-related death defined as death that was not caused by any underlying or contributing cause of death with ICD-10 code: 'A', 'B', 'U'. Patients who die from these causes are censored at the date of death. | | | | | | | | | | | | |
